# Modern Emerging Biosensing Methodologies for the Early Diagnosis and Screening of Ovarian Cancer

**DOI:** 10.3390/bios15040203

**Published:** 2025-03-21

**Authors:** Farah Abul Rub, Naseel Moursy, Nouf Alhedeithy, Juraij Mohamed, Zainab Ifthikar, Muhammad Affan Elahi, Tanveer Ahmed Mir, Mati Ur Rehman, Saima Tariq, Mubark Alabudahash, Raja Chinnappan, Ahmed Yaqinuddin

**Affiliations:** 1 College of Medicine, Alfaisal University, Riyadh 11533, Saudi Arabia; fabulrub@alfaisal.edu (F.A.R.); nmoursy@alfaisal.edu (N.M.); nalhedeithy@alfaisal.edu (N.A.); zifthikar@alfaisal.edu (Z.I.); melahi@alfaisal.edu (M.A.E.); tmir@kfshrc.edu.sa (T.A.M.); 2 Faculty of Medicine, University of Colombo, Colombo 00800, Sri Lanka; juraij7777@gmail.com; 3 Laboratory of Tissue/Organ Bioengineering & BioMEMS, Organ Transplant Centre of Excellence (TR&I-Dpt), King Faisal Specialist Hospital & Research Centre, Riyadh 11211, Saudi Arabia; 4 Department of Biological and Biomedical Sciences, The Aga Khan University, Stadium Road, P.O. Box 3500, Karachi 74800, Pakistan; mati.rehman@aku.edu; 5 Department of Obstetrics and Gynecology, Al Iman General Hospital, Ministry of Health, Riyadh 12684, Saudi Arabia; smohammed@moh.gov.sa; 6 Strathclyde Institute of Pharmacy and Biomedical Sciences (SIPBS), Glasgow G4 0RE, UK; mubark.alabudahash@strath.ac.uk

**Keywords:** ovarian cancer (OC), biosensors, cancer biomarkers, cancer diagnosis

## Abstract

Ovarian cancer (OC) is one of the leading causes of gynecological cancer-related death worldwide. Late diagnosis at advanced stages of OC is the reason for a higher mortality rate. Earlier diagnosis and proper treatment are important for improving the prognosis of OC patients. Biosensors offer accurate, low-cost, rapid, and user-friendly devices that can be employed for the detection of OC-specific biomarkers in the early stage. Therefore, it is important to consider the potential biomarkers in the biological fluids to confirm the OC prognosis. Out of many biomarkers, the most commonly tested clinically is cancer antigen 125 (CA-125). However, CA-125 is considered to be a poor biomarker for OC diagnosis. Several biosensing methods were developed for the sensitive and quantitative detection of each biomarker. In abnormal expression in OC patients, nucleic acids, enzymes, cells, and exosomes are used as target biomarkers for the construction of biosensors. This review focuses on the development for the detection of various biomarkers using multiple biosensing methods. Here, we describe the origin and the significance of OC-associated biomarkers, the working principle of biosensors, and the classification of biosensors based on their recognition elements and signal transducers. The modes of detection and sensitivity of the sensors are discussed. Finally, the challenges in the fabrication, obstacles in the clinical application, and future prospects are discussed.

## 1. Introduction

**Figure 1:**
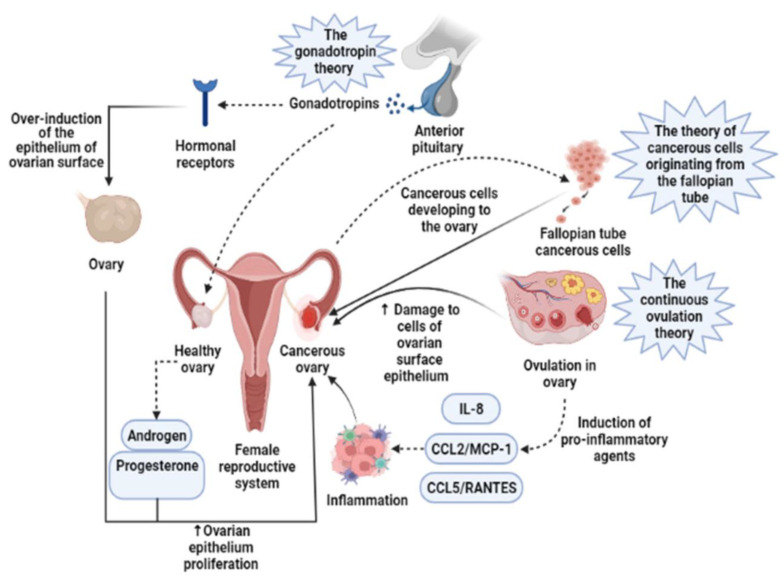
Three main theories regarding the development of ovarian cancer are based on induction of the epithelium of the ovarian surface by hormonal receptors, increased induction of pro-inflammatory agents during continuous ovulation, and cancerous cells originating from the fallopian tube. IL-8, Interleukin-8; CCL2/MCP-1, Monocyte chemoattractant protein-1; CCL5/RANTES, CC Chemokine Ligand-5. The image is adopted from Tazangi et al., 2021, with copyright permission under the terms of the CC BY NC ND 4.0 license [12].

Ovarian cancer (OC) is a significant public health challenge, accounting for a substantial proportion of cancer-related deaths globally [1]. OC is a prevalent reproductive organ malignant tumor in women, often diagnosed at the advanced stage. It is susceptible to spreading in the pelvic and abdominal cavities, resulting in malignant ascites [2]. This is partly due to its aggressive nature and the fact that it is usually diagnosed in later stages, portending poor outcomes. Its underlying etiopathogenesis involves a complex interplay of genetic, hormonal, and environmental factors contributing to its development and progression. OC has three main subtypes: epithelial (most common), sex-cord-stromal, and germ cell, with the latter two accounting for about 5% of all OCs [3]. Epithelial ovarian cancer is further divided into four primary histologic subtypes: serous, endometrioid, clear cell, and mucinous [3]. Development of OC by the induction of the epithelium of the ovarian surface is represented in Figure 1. Serous tumors are of two types, high-grade serous carcinomas (HGSCs) or low-grade serous carcinomas (LGSCs). Simulation studies suggest that earlier detection of preclinical OC may improve survival rates by 10–30% while also being cost-effective [4]. The current detection method involves transvaginal ultrasound and CA-125 analysis. Although CA-125 is a widely used biomarker, its specificity is limited because of its association with the menstrual cycle, pregnancy, and overexpression during inflammation, as well as in other gynecologic disorders, e.g., endometriosis [5]. Conventional methods, such as polymerase chain reaction (PCR) assay, radioimmunoassay, electrophoretic immunoassay, enzyme-linked immunosorbent assay (ELISA), and mass spectrometric immunoassay, are frequently used for the diagnosis. All these methods require prewashing, separation, and concentration steps [2]. Despite the use of highly expensive bulky instruments and trained professionals, early-stage diagnosis and therapy are still not very efficient. In addition, these processes require a hefty amount of time for the analysis of the sample. Therefore, a simple, highly sensitive, rapid, and low-cost analytical device is required for the point-of-care testing. Biosensors have become an important state-of-the-art, modern analytical technology that plays a significant role in the clinical diagnosis of the patient and timely treatment. Detection of biomarkers in various types of diseases including cancer have been described [6,7,8]. This review highlights the overview of the biomarkers associated with OC and their diagnosis by modern biosensing methodologies, including optical, electrochemical, fluorescence-based, FRET-based, electrochemiluminescence, SPR, and colorimetric biosensing, which have been used for cancer diagnosis [9,10,11]. This has prompted further research into this area. This review aims to summarize and provide an overview of the significant role of the current biomarkers that have been and are being investigated for the early detection of ovarian cancer.

### 1.1. CA-125

CA-125, also known as MUC16, is a protein encoded by the MUC16 gene, has been a widely adopted tumor marker for ovarian cancer over the past 30 years, and has been employed to diagnose OC, as well as to measure its prognosis [13,14]. Bast and colleagues first introduced CA-125 in 1981 by developing a monoclonal antibody against CA-125 antigen [15]. Around 80% of women diagnosed with advanced-stage epithelial ovarian cancer have elevated levels of serum CA-125 [16]. However, CA-125 has limited specificity in the detection of early-stage OC; only about half of early-stage OC patients present with elevated serum CA-125 [17]. Furthermore, several comorbidities can also lead to an elevation in serum CA-125 levels, e.g., hepatic cirrhosis, endometriosis, normal menstrual cycles, uterine fibroids, and pelvic inflammatory disease. This manifests that CA-125 lacks the necessary specificity and sensitivity to be used as a dependable biomarker for early-stage OC detection [18].

### 1.2. HE4

Human Epididymis Protein 4 (HE4) is a whey acidic four-disulfide core (WFDC) protein, first identified in the distal epididymal epithelium [19]. HE4 expression is linked to cancer cell adhesion, migration, and tumor proliferation via its effects on the EGFR-MAPK pathway [20]. A study by Costa et al. demonstrated that while HE4 is not expressed by the normal ovarian surface epithelium, it is expressed in all cases of human endometrioid epithelial ovarian cancers, and 93% of serous ovarian carcinomas stained positive for HE4 [21]. Studies have demonstrated that when combined with CA-125, it provides greater specificity than CA-125 alone. HEP4 also has a higher sensitivity for detecting OC compared to CA-125, which could be due to its resistance to interference from benign pelvic disease [5]. An ELISA analysis of serum HE4 levels in 37 OC patients, by Schummer et al., revealed that when compared to 65 healthy controls, HE4 exhibited comparable sensitivity and specificity to serum CA-125 whilst having fewer false positives in individuals without OC [22]. It is noteworthy that HE4 is significantly elevated in both ovarian and endometrium cancer, but not in endometriosis.

### 1.3. Human Prostasin (PSN)

Human prostasin (PSN) is a glycosyl-phosphatidyl-inositol (GPI)-anchored extracellular serine protease. It is encoded by PRSS8, which is located on chromosome 16p11.2. It is involved in the activation of epithelial sodium channels, as well as in the inhibition of in vitro invasive prostate and breast cancer [23]. Altered expression of prostasin is associated with multiple cancer types, e.g., urinary, uterine, prostatic, and ovarian, when compared to levels in corresponding normal tissue. Mok et al. proposed the use of prostasin as a biomarker for ovarian carcinoma by using microarray technology to identify upregulated genes associated with secretory proteins [24]. PSN is overexpressed in malignant ovarian cells and stroma when compared to healthy ovarian tissue, with a specificity of 94% and a sensitivity of 51.4% [25]. Ahmed et al. have demonstrated that PSN overexpression is not only present in early-stage OC but is retained across higher stages and grades [26].

### 1.4. Mesothelin

Mesothelin is a tumor differentiation antigen that is expressed in most ovarian epithelial cancers and is an emerging biomarker for OC [27,28,29]. The interaction between mesothelin and CA-125 not only aids in cancer cell adhesion to the mesothelial peritoneal epithelium but may also play a role in peritoneal metastasis of OC [30,31]. A study by Badgewell et al. demonstrated the presence of mesothelin in both the urine and serum of patients with early-stage OC [32].

### 1.5. Osteopontin (OPN)

Osteopontin is a glycoprotein with adhesive properties, secreted by activated T lymphocytes, macrophages, and leukocytes. It is present in the extracellular matrix, at sites of inflammation as well as in various body fluids. OPN is expressed not only in OC but also in endometrial, cervical, colorectal, breast, non-small cell lung cancer, prostate, hepatocellular, and gastric cancers. A study by Schorge et al. demonstrated that OPN is clinically inferior in predicting response to chemotherapy but was elevated in early-stage OC [33].

### 1.6. Kallikreins

Kallikreins are a subgroup of serum proteases that have various physiologic roles. They are also involved in processes critical to cancer progression, e.g., proteolysis, signal transduction, and cellular proliferation [34]. They are expressed in endocrine and epithelial tissues, which are regulated by hormones in cancer and are detected in human body fluids. Studies have demonstrated that certain kallikreins, notably, KLK5, KLK6, and KLK10, can be identified in the ascitic fluid of patients with ovarian cancer, with average concentrations measured at 62.2 ng/mL, 144 ng/mL, and 57 ng/mL, respectively [35]. Moreover, elevated levels of kallikreins portend a poorer prognosis in ovarian cancer [36].

### 1.7. Mucin 1 (MUC1)

Mucin 1 (MUC1) is a transmembrane glycoprotein implicated in ovarian cancer biology. Aberrant expression of MUC1 has been noted in a majority of epithelial ovarian cancers, with studies indicating overexpression in 90–100% of serous carcinomas [37,38]. This overexpression usually heralds poor clinical outcomes, along with increased invasiveness and chemoresistance, rendering MUC1 a critical target for therapeutic strategies [39,40]. Interestingly, autoantibodies against aberrantly glycosylated MUC1 have been associated with ovarian cancer, and they have the potential to serve as non-invasive biomarkers for earlier diagnosis of OC [41].

### 1.8. Heat Shock Proteins (HSPs)

HSPs, owing to their roles in tumor biology, e.g., cell survival, proliferation, and response to therapy, have garnered significant attention as potential biomarkers for OC. HSPs, especially HSP70, HSP27, and HSP90, have been found to be overexpressed in many malignancies, including OC, and the expression levels appear to correlate with disease progression and prognosis [42,43,44]. HSP27 has been associated with advanced disease stages and peritoneal metastasis of OC [43]. Bodzek et al. have demonstrated that antibodies against HSP60 and HSP65 are detected in the sera of women with OC, and detection of these antibodies could provide a non-invasive method of early diagnosis and monitoring of OC [42].

### 1.9. MicroRNAs (miRNAs)

Circulating acellular miRNAs have been identified as potential clinical indicators for cancer, including OC, and distinct profiles have been shown to correlate with disease presence and progression [45,46,47]. Moreover, dysregulation of miRNAs is often associated with acquired chemoresistance in OC. The epigenetic silencing of miR-199b-5p has been linked to the activation of the JAG1-Notch1 signaling pathway, which underlies OC cell chemoresistance [48]. Furthermore, miR-27b-5p regulates the growth and metastatic behaviors of OC cells by targeting CXCL1 and contributing directly to tumor aggressiveness [49]. Similarly, miR-665 targets SRCIN1 and thereby promotes OC cell proliferation [50]. Recent developments in liquid biopsy have facilitated the identification of circulating miRNAs, such as miR-99a-5p and miR-145-5p, as potential OC biomarkers [51,52]. MicroRNAs (miRNAs) are seen as the main future biomarker for managing ovarian cancer [53]. miRNA has aberrant expression in tumor development; it is a potential diagnostic, prognostic, and predictive tool for OC, while also regulating it. Interestingly, miRNAs are being explored for their therapeutic value, owing to the fact that they impact most pathways of carcinogenesis, like angiogenesis, epithelial–mesenchymal transition, alterations in extracellular matrix biology, cancer cell proliferation, invasion, metastasis, response, and chemosensitivity to certain drugs. However, studies in this domain are still limited [54].

### 1.10. Exosomes

Exosomes are small extracellular vesicles that are secreted by various cell types and are used as potential biomarkers for cancer detection [55]. Recent studies have demonstrated that exosomal miRNAs can serve as biomarkers for OC. For example, elevated levels of circulating miR-205 exosomes have been linked to increased metastasis and angiogenesis in ovarian cancer patients, rendering it as a potential diagnostic and prognostic marker [56]. Potential use of exosomes as biomarkers extends beyond miRNAs to include proteins and other molecules [55,57]. For example, annexin A3 has been identified in exosomes from platinum-resistant OC cells [58]. Moreover, exosomal proteins such as CRABP2 are upregulated in OC and correlate with enhanced cellular proliferation, highlighting their potential as diagnostic markers [59].

## 2. Biosensors

**Figure 2:**
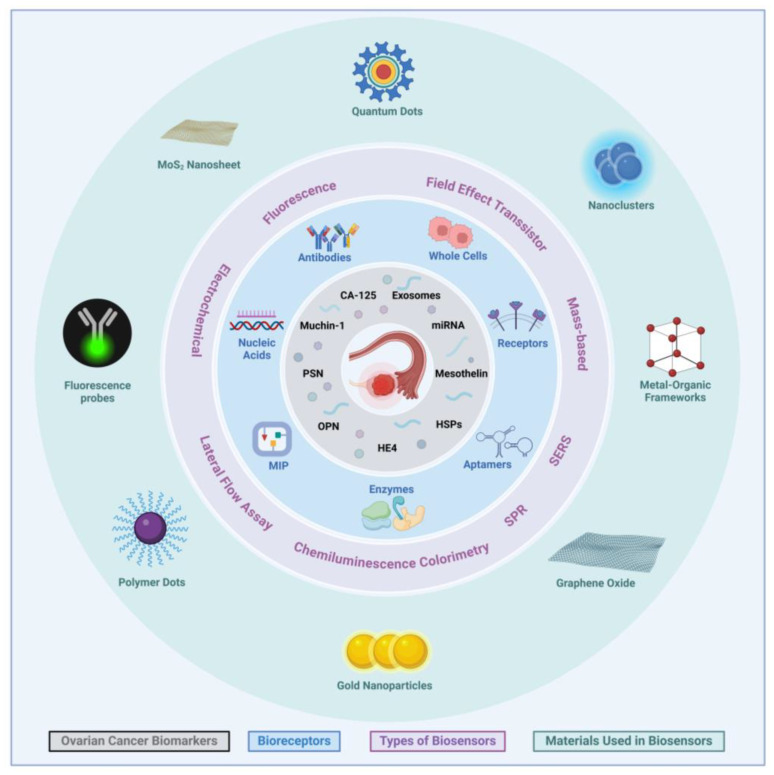
Schematic representation of ovarian cancer biomarkers detection using various recognition elements and sensing materials used for sensor development.

Conventional assays for the detection of cancer, particularly CA-125, include ELISA, Radioimmunoassay(RIA), and Fluorescence immunoassay (FIA), are not cost-effective, are tedious to perform, and require trained operators [60]. In contrast, the use of biosensors in cancer diagnosis created an avenue for efficient and easier point-of-care diagnosis. This could potentially allow for early disease detection and treatment, providing better monitoring of treatment efficacy and an overall lower mortality rate [61]. Biosensors have been employed in numerous industries, including in diagnostics such as for cancer, using biomarkers [62]. Recent studies have used nanomaterials in manufacturing biosensors, which have allowed them to become mobile, sensitive, and efficient [10,63]. Biosensors can be classified by several themes, based on their biorecognition element, transduction mechanism, or detection system [63]. This review probes the transduction classification, which comprises aspects such as optical, electrochemical, mass-based, paper-based, and electronic biosensors. Another way to classify biosensors could be based on biomolecular labeling. Traditional biosensors use fluorescence labels to detect substances. However, due to the tedious labeling process, increased costs, and low sensitivity, they have been replaced by label-free options that utilize the unique refractive index of molecules for an optical detection method [64]. Detection of various OC biomarkers recognized by recognition receptors and materials applied for the different detection methods are illustrated in Figure 2.

### 2.1. Optical Biosensors

#### 2.1.1. Fluorescence Sensors

One of the most important methods of optical biosensors is fluorescence-based detection. This is evident in the experiment of Wu et al., which focuses on the implementation of graphene oxide nanomaterial and its benefit in the advancement of the diagnosis of ovarian cancer. The experiment designs a microfluidic fluorescence biosensor from a combination of a microfluidic detector and a capturing antibody-immobilized microfluidic chip with a graphene oxide layer. Afterward, samples with the targeted biomarkers are added to the biosensor, and a reaction occurs among immobilized antibodies, leading to a high fluorescent intensity that is dependent on the concentration of the added biomarker. In this method, they detect four different biomarkers, such as CA-125, HE4, CEA, and APF, simultaneously. The biosensors demonstrated the LOD of 0.01 U/mL for CA-125 and ~1 pg/mL for HE4, CEA, and APF, respectively, signifying their role in the early screening of ovarian cancer [65]. Abou-Omar et al. developed gold nanoparticles (AuNPS) with a thin sol–gel film coated by a Schiff base ligand. Its optical performance was evaluated through the quenching effect, where protic solvents have high fluorescent intensity and more sensor stability. On the other hand, aprotic solvents are unstable, resulting in fluorescent intensity quenching. This work demonstrates a linear relationship between cancer antigens and fluorescent light emission, depicting the non-invasiveness and simplicity of fluorescent-based biosensors with high efficiency. The sensors were validated using patients diagnosed with ovarian cancer. The detection range of 2.0–127.0 U/mL for CA-125 was observed, with an LOD of 1.45 U/mL [66].

As an illustration, De La Franier & Thompson utilized fluorescence spectroscopy to identify lysophosphatidic acid (LPA), a signaling lipid that could serve as a potential biomarker found in all stages of ovarian cancer, thereby facilitating early screening [67]. In developing this biosensor, they discovered that the protein gelsolin binds to lysophosphatidic acid (LPA) through the PIP2-binding domain, acting as a selective probe with a high affinity for LPA. Furthermore, gelsolin, being an actin-binding protein, interacts with actin at three different sites, indicating that LPA regulates the binding between gelsolin and actin. This suggests that the actin–gelsolin combination is well suited for detecting LPA through fluorescence spectroscopy. To employ this biosensor, dyed actin is initially pre-tagged with signaling molecules for measurement. It is then attached to a solid surface containing gelsolin. Following this step, the dyed actin is released into a liquid sample in the presence of LPA. The dye concentration in the liquid sample correlates with the concentration of actin, and from its measurement, the concentration of LPA can be determined. This biosensor test, utilizing a dual-protein system of actin and gelsolin, allows for surface deposition into silica nanoparticles. Moreover, incorporating rhodamine dye into actin generates a fluorescence signal free from LPA interference, thus proving its benefit in the rapid diagnosis of all stages of ovarian cancer, with an LOD of 5 µM LPA in serum [68].

In addition, to further emphasize the increased utilization of fluorescence spectroscopy in the field of biosensors, Attia et al. established a spectrofluorometric assay for the detection of the CA-125 biomarker based on the luminescence intensity quenching of phthalocyanine fluorophore to diagnose ovarian cancer. Phthalocyanines (Pcs) are macrocyclic compounds that absorb and emit light, with only a few functioning in the near-infrared region (NIR), such as non-peripheral substituted phthalocyanines. Therefore, this biosensor consists of a phthalocyanine–polystyrene film, synthesized with near-infrared region phthalocyanines as NIR fluorescent dye inserted into a polystyrene matrix, forming a rigid, impenetrable polymer thin film. While obtaining human serum samples for the testing of ovarian cancer, to avoid the presence of other biomarkers like CEA and CA-15-3, along with CA-125, each serum sample is placed on a plate covered with an antibody specific to CA-125. Afterward, it is washed with phosphate buffer to ensure the isolation of the needed biomarker and minimize interference from others. The optical sensor performance is initiated when nickel is added to the near-infrared-region phthalocyanines as they are embedded into a polystyrene matrix (NiPc@PS) thin film [69]. This demonstrates interaction with samples containing CA-125 through the monitoring of luminescence signal quenching, thus providing a wide linear range of concentration from 1.0 × 10^−2^ to 127 U/mL and the LOD of 1.0 × 10^−4^ U/mL [69].

To demonstrate other uses of fluorescence, Bahari et al. incorporated the high selectivity of magnetic molecularly imprinted polymers (MMIPs) into the high-sensitivity fluorescence procedure. Their work introduced effective and inexpensive nanoclusters such as nickel (Ni NCs) and Nobel Cd (Cd NCs). The emphasis was on building a multiplex fluorescence system to differentiate signals produced by multiple biomarkers for simultaneous measurement. The MMIPs were prepared through magnetic graphene oxide (GO–Fe_3_O_4_), and the new fluorescent luminophore was made from Ni NC- and Cd NC-topped bovine serum sample albumin (BSA). Later on, the CA-125 and CA-15-3 antibodies from human samples were incubated in the solution, representing ovarian cancer and breast cancer biomarkers, respectively, revealing that the fluorescent light emission of the Ni NCs and Cd NCs strengthened as the concentration of biomarkers in the sample increased. This fluorescence–MMIP immunosensor has excellent stability and permits immobilization of antibodies in the concentration range of 0.0005–40/mL with an LOD of 50 μU/mL, allowing its implementation in other aspects of clinical research [70].

Förster resonance energy transfer (FRET) quenching, is a form of dynamic quenching, where energy is passed from the fluorescent donor or reporter dye to the quencher without any light being absorbed or emitted. Biosensors utilizing FRET are being developed for the targeted detection of biomolecules in multiple biomedical fields [71]. One example of its application is a study by Omer et al., which utilized carbon quantum dots (CDs) produced from ortho phenylenediamine instead of the usual QDs that illuminate under ultraviolet light. The optical properties of CDs are highlighted after embedding them in a polymethyl methacrylate (PMMA) matrix, illustrating a thin fluorescent film. Consequently, it exhibited illuminance through multiple colors. This technique, specifically, in detecting ovarian cancer, relies on quenching. Once the CDs bind to CA-125, an electrostatic reaction is formed, yielding a nonfluorescent complex controlled by the concentration of CA-125 in the range of CA-125 from 0.01 to 129 U/mL, with an LOD of 0.66 U/mL [72]. Therefore, it rapidly provides a valid and accurate visual biomarker measurement.

**Figure 3:**
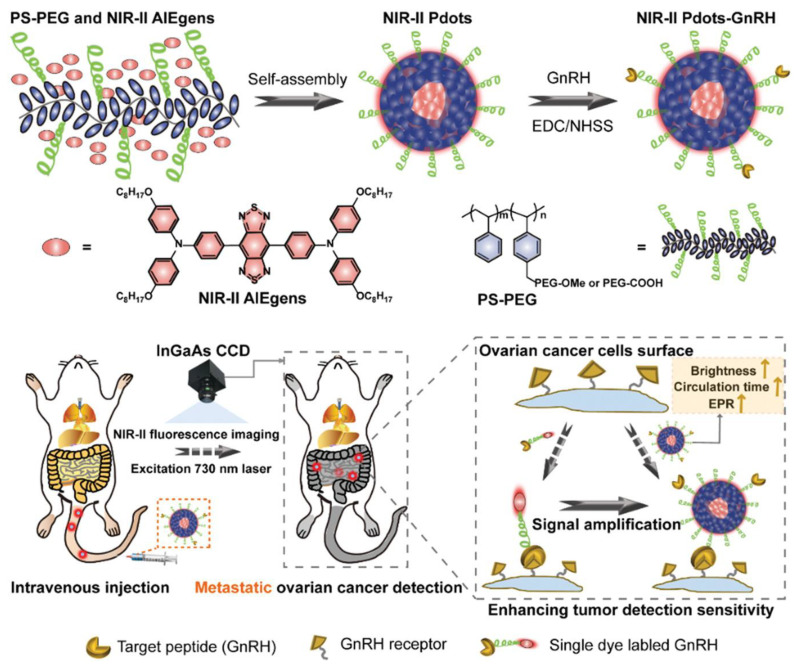
Schematic illustration for preparation of tumor-targeted NIR-II Polymer dots and their application for in vivo metastatic ovarian cancer detection. This image is adapted from the original Zhou et al., 2021, with copyright permission under the terms of the CC-BY-NC-ND 4.0 license [73].

Moreover, for intraoperative diagnosis of ovarian cancer, fluorescence imaging is the preferred choice. Zhou et al. established NIR-II fluorescence imaging with polymer dots (NIR-II Pdots), particularly to target metastasis and enable early diagnosis. To implement this design, the NIR-II Pdots must self-assemble based on hydrophobic interaction with water, where NIR-II aggregation generates emission luminogens (AIEgens) within a polystyrene-graft-polyethylene glycol (PS-PEG) matrix to prolong blood circulation time. The modification of NIR-II Pdots with the insertion of peptides, such as tumor-specific cetrorelix, forms NIR-II Pdots-GnRH, which target gonadotropin-releasing hormone receptors that are overexpressed in ovarian cancer, heightening affinity towards the tumor cells [73]. AIEgens exhibit strong fluorescence, which is further enhanced by mixing tetrahydrofuran (THF) with methanol. Further, NIR-II Pdots have brighter probes compared to other nanoparticles in the aggregate state. To observe the effects of Zhou et al.’s strategy, intravenous injection of the NIR-II Pdots enables the visualization of hematogenous and lymphatic metastasis, enhancing real-time detection in NIR-II fluorescence imaging (Figure 3).

**Figure 4:**
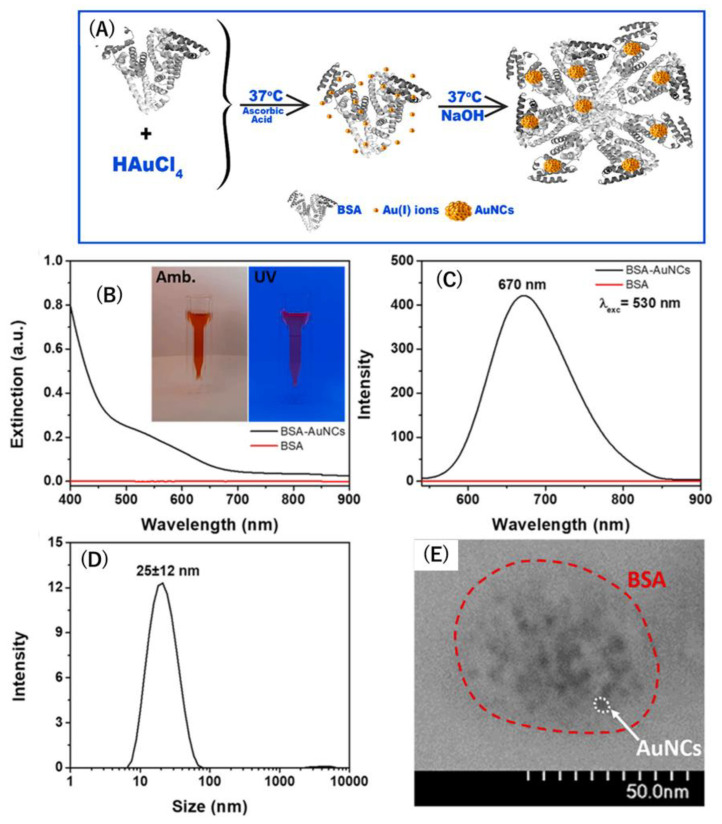
(**A**) Schematic representation of the two-step synthesis of photoluminescent BSA-AuNCs. (**B**) The absorption spectra (the inset—BSA-AuNCs under ambient (amb.) and UV illumination); (**C**) the hydrodynamic size of BSA (red line) and BSA-AuNCs (black line); (**D**) the photoluminescence; and (**E**) the HRTEM image of BSA-AuNCs. The image is adapted from Hada et al., 2021, with copyright permission [74].

With extensive research conducted on photoluminescent gold nanoclusters, Hada et al. demonstrated bovine serum albumin-stabilized gold nanoclusters (BSA-AuNCs) with photoluminescent emission to enhance comprehension of the topic. Consisting of protein-polymerized chains submerged in AuNCs with BSA covalently bound to folic acid, which is strongly associated with folate receptor α (FRα), this protein is depicted in epithelial ovarian tumors. The addition of folic acid enabled Hada et al. to pioneer a label-free contrast agent for visualizing NIH: OVCAR-3, an ovarian cancer epithelial cell line, through fluorescence imaging. Specifically, this involved incorporating fluorescence lifetime imaging microscopy (FLIM) of higher quality compared to traditional fluorescence imaging. In this method, NIH, OVCAR-3 cells marked with AuNCs confirmed the uniform aggregation of FA-BSA-AuNCs, suggesting greater cellular uptake compared to BSA-AuNCs alone. This facilitates diagnosis and potentially guides treatment. To link proteins with preformed AuNCs, BSA contains stabilized functional thiol groups on cysteine residues, emitting a bright-red color under ultraviolet light, highlighting the photoluminescent effect (Figure 4) [74]. Many advantages of the photoluminescent BSA-stabilized AuNCs functionalized with FA for targeted fluorescence imaging were presented. These include the utilization of AuNCs as nanothermometers due to their temperature sensitivity and the achievement of more specific detection of ovarian cancer through the incorporation of folic acid.

#### 2.1.2. Optical Aptasensors

Aptasensors are types of biosensors where aptamers are used as recognition elements for the rapid and sensitive detection of analytes. Aptamers are single-stranded DNA or RNA that specifically recognize the target with high affinity and sensitivity. Aptamers are highly stable compared to antibodies. They are used for the construction of biosensors for a wide range of targets, from small molecules to large proteins [75,76,77,78]. A well-known method for analysis is chemiluminescence biosensors, which rely on the chemical reaction between a biological recognition element and the target analyte, emitting light for concentration measurement [79]. Therefore, Han et al. developed a dual-aptamer-conjugated biorecognition chemiluminescence aptasensor for the detection of carcinoembryonic antigen (CEA) for ovarian cancer diagnosis. Monitoring carcinoembryonic antigen (CEA) is crucial in high-risk individuals and should be utilized in screening. This chemiluminescence aptasensor incorporates metal–organic frameworks (MOFs) due to their large surface area, which facilitates efficient immobilization of bioreceptors interacting with the analyte. Additionally, MOFs enhance the sensitivity and selectivity of biosensors. The MOF in this example is a metalloporphyrinic iron-based compound called hemin@MIL-88B (Fe), functionalized with the luminol anion and conjugated with aptamers apt1 and apt2, respectively. These aptamers undergo conformational changes to self-assemble, forming hemin@MIL-88B (Fe)-apt1/CEA/L-apt2 sandwich-like ternary complexes that recognize CEA. The single-stranded DNA (ssDNA) is immobilized on the surface of the Fe_3_O_4_@SiO_2_ magnetic material, with the complementary bases of the ssDNA allowing for the absorption of hemin@MIL-88B aptamers onto the magnetic carbon nanotubes (MCNTs). With chemiluminescence at play, the intensity exhibits a linear relationship with CEA concentration [80]. The utilization of materials such as hemin@MIL-88B (Fe), possessing higher affinity and catalytic performance compared to solely hemin or MIL-88B (Fe), makes the dual-aptamer-conjugated biorecognition chemiluminescence aptasensor feasible, stable, and reproducible. Under the optimal conditions, this method detects CEA in the range of 0.01–100 ng/mL, with an LOD of 1.5 × 10^−3^ ng/mL. This allows for the simultaneous detection of various cancers, such as ovarian cancer, through CEA [80].

#### 2.1.3. Colorimetric Biosensors

Researchers commonly use colorimetric biosensors, a simple and easy approach that visualizes the interaction between the analyte and probes through color changes [81]. Hasan et al. introduced a colorimetric nano-biosensor featuring gold nanoparticles (AuNPs), designed for the detection of platelet-derived growth factor (PDGF), a biomarker linked to ovarian cancer. PDGF facilitates the growth of ovarian epithelial cells, thus playing a role in the progression of ovarian tumors by promoting the vascularization of the cells. To initiate this procedure, PDGF-specific aptamers are attached to AuNPs, enabling the formation of multiple chemical bonds and preventing digestion by nucleases. Subsequently, aggregation of AuNPs occurs upon the absorption of aptamers, leading to a color change along the dispersed particles. In Hasan et al.’s approach, the colorimetric action occurs without the need for any instrument; it is detectable by the naked eye. The color shifts from pink to light purple as the concentration of PDGF increases, leading to enhanced aggregation of AuNPs. The colorimetric nano-biosensor, designed to detect platelet-derived growth factor (PDGF) through the implementation of AuNPs, is easy to operate and cost-effective. The PDGF was tested in the range of 0.01–10 μg/mL, with an LOD of 0.01 μg/mL. It aids in accurately diagnosing ovarian cancer and holds promise as a potential early screening tool [82].

**Figure 5:**
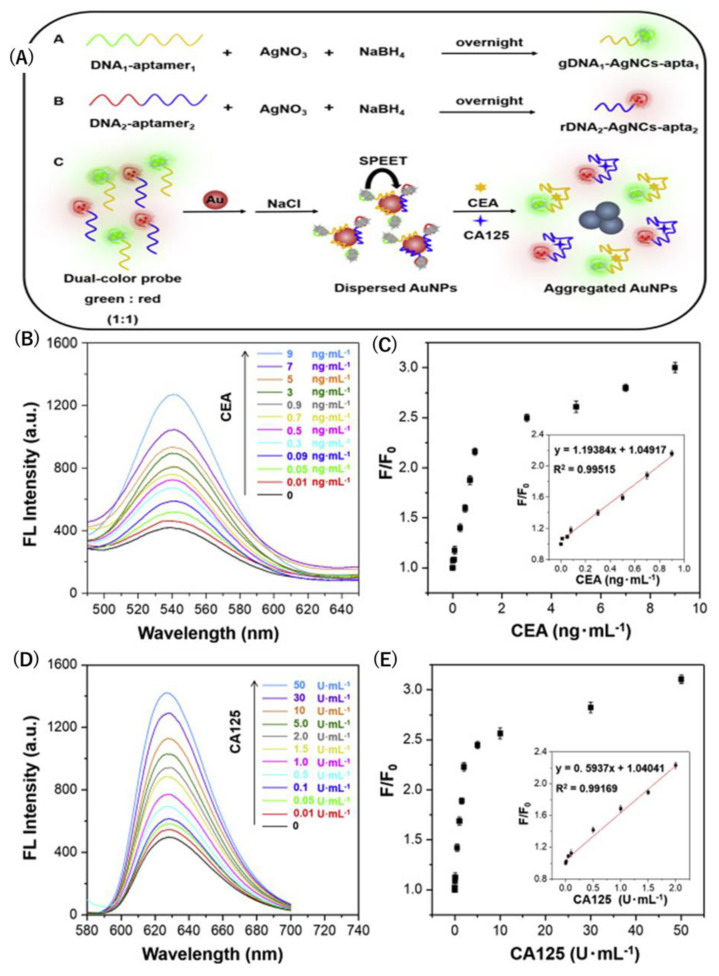
(**A**) Schematic illustration of the interaction between DNA-AgNCs-aptas and AuNPs in the absence and in the presence of CEA and CA-125, and the corresponding fluorescence responses. (**B**,**D**) Evolution of fluorescence spectra of gDNA1-AgNCs-apta1 and rDNA2-AgNCs-apta2 (in the presence of AuNPs and NaCl) with the increase in target amount, respectively. (**C**,**E**) The linear relationships between F/F0 and the concentrations of CEA and CA-125, respectively. F/F0 represents the fluorescence intensity in the presence and absence of targets. The image is adapted from Xu et al., 2020, with copyright permission [83].

Furthermore, Xu et al. proposed a strategy of fluorescent quenching based on surface plasmon-enhanced energy transfer (SPEET) for the early diagnosis and monitoring of ovarian cancer. This new strategy includes a salt-induced gold nanoparticles (AuNPs) dual fluorescent aptasensor based on DNA-AgNCs, which have high affinity to targeted locations in a DNA sequence. These aptamers (DNA-AgNCs-aptas) cause a reduction of silver (Ag) ions in the presence of specified DNA oligonucleotides. When AuNPs are present in a high-salt concentrated solution, DNA-AgNCs-aptas are absorbed, and the fluorescent quenching mechanism based on SPEET takes place, hindering the aggregation of salt-induced AuNPs. However, as the CEA and CA-125 biomarkers are incubated, they bind with the analytes, inducing a decline in SPEET capability, in which AuNPs aggregation happens with the retrieval of fluorescent DNA-AgNCs-aptas. The separation of DNA-AgNCs-aptas from the surface of AuNPs generates a dual light emission from DNA-AgNCs-aptas: green with CEA aptamer and red with CA-125 aptamer. The LODs for CEA and CA-125 were 7.5 pg/mL and 0.015 U/mL, respectively (Figure 5). With a one-to-one correspondence between the fluorescence light ratio and biomarker concentration, the dual-color fluorescent aptasensor can be noted as reliable, accurate, and sufficient in meeting clinical demands [83].

#### 2.1.4. Surface Plasmon Resonance (SPR) Biosensors

Another method, surface plasmon resonance (SPR), has attracted significant research interest. This method is a real-time label-free optical biosensor that targets the desired biomolecules, without the need for fluorescence. Its integration in ovarian cancer diagnosis is apparent in Yi et al.’s study, which has delved deeply into the topic by comparing the two most commonly used films in SPR detectors: gold and silver. The study used a combination of gold–silver alloy and a film-based SPR (AuAg-SPR) sensor to attain the high chemical stability of gold and the high sensitivity of silver. Based on the principle of measurement using the refractive index (RI), the resonance wavelength and light intensity change as biomolecules are absorbed onto the SPR chip surface. The results showed that the AuAg-SPR sensor had a higher sensitivity and a hundred times lower limit of detection than that of the gold SPR sensor alone (Au-SPR) for CA-125. The sensor was tested in the concentration range of 0.1 to 10 U/mL, and the LOD of the sensor was 0.1 U/mL (0.8 ng/mL). Significant outcomes of this invention will play a major role in the future of biomarker detection research [84]. In addition, Liu et al. created an intensity-modulated compact SPR sensor that consists of conventional SPR without the nanostructure fabrication to eliminate the expensive costs and allow for feasibility. This new portable SPR device is adaptable to various clinical applications and has demonstrated higher detection sensitivity for exosomal proteins than ELISA [85].

As an extension of the series of studies around SPR biosensors, a new approach was developed: surface plasmon resonance imaging (SPRi). Instead of using traditional sensorgrams to record surface plasmon resonance signals, SPRi takes it a step further by converting these signals into images captured through a CCD camera. For instance, Szymanska et al.’s paper introduces a non-fluidic array version of the SPRi approach for determining the HE4 biomarker in ovarian cancer diagnosis. The non-fluidic array version does not require signal attenuation to achieve the necessary limit of quantification (LOQ) or preparatory preconcentration of the analyte for its multiplexing capability. This biosensor consists of a gold chip coated with cystamine, which is then linked to an antibody against HE4 through an amine group. An SPRi signal was measured after immobilization with the antibody to document the interaction, signifying the presence of the HE4 biomarker. Szymanska et al.’s label-free and fast-acting biosensor approach serves as a powerful diagnostic tool that can detect HE4 in the range of 2–120 pM, with an LOD of 2 pM [86]. This situation has enabled Szymańska et al. to propose an SPRi biosensor specific for circulating CA-125/MUC16, biomarkers for ovarian cancer. The proposed biosensor consists of the same gold chip covered with photopolymers and hydrophobic paint, for concurrent documentation of the results without mixing the solutions. To facilitate immobilization of the antibody and interaction with the biomarkers, the activated receptor was placed on a thiol (cysteamine)-modified surface and then incubated. Next, bovine serum albumin (BSA) in phosphate-buffered saline (PBS) was applied onto the chip to minimize interference from other compounds and their influence on results. This denotes high selectivity and a proportional relationship to CA-125/MUC16, with an LOD of 0.66 U/mL [87].

Since its introduction, more studies have been conducted on surface plasmon resonance imaging (SPRi), leading to increased usage of SPRi and demonstrating its widespread growth and implementation in the field of biosensors. For instance, Oldak et al. proposed two analytically specific SPRi biosensors for measuring the levels of cathepsin S in diagnosing ovarian cancer, thereby solidifying the role of cathepsin S as an ovarian cancer biomarker. The first SPRi biosensor proposal is based on antibody interaction, wherein an antibody against cathepsin S is present in the sensor. This antibody can be immobilized through a cysteamine linker, which coats the gold chip for direct measurement of the specified biomarker. On the other hand, the second SPRi biosensor is based on inhibitor interaction, wherein LY3000328, a specific inhibitor for cathepsin S, was selected and laid onto active sites in the biosensor. In this design, the gold chip was covered with a 1-octadecanothiol linker to facilitate hydrophobic interaction with the cathepsin S inhibitor, resulting in its immobilization. Next, to obtain the measurements for diagnosis through the SPRi signal, an image is captured when the antibody or inhibitor is immobilized on the biosensor surface. Following this, an interaction with the cathepsin S-containing sample occurs. After that, it is washed with HBS-ES buffer to prevent non-specific absorption, and another image is taken [88]. Hence, SPRi signals quantify the concentrations of the cathepsin S biomarker, considering the high concentrations detected in ovarian cancer patients. Despite several articles discussing SPRi biosensors for the detection of cathepsins B, D, E, G, and L, none have focused on cathepsin S. This signifies that Oldak et al. were the first to develop this analytically specific model, exhibiting high precision and sensitivity, with an LOD of 0.04 ng/mL.

#### 2.1.5. Surface-Enhanced Raman Spectroscopy (SERS)-Based Biosensors

Recent studies have shown successful implementations of surface-enhanced Raman spectroscopy (SERS)-based immunosensors in cancer biomarkers detection. SERS is a technique based on light scattering after it strikes a nanostructured metallic surface. The intensity of the incident light signal indicates the concentration of the targeted biomarker. In particular, for the detection of a protein cancer marker, a combination of immunoassay and surface-enhanced Raman scattering (SERS) with an immunoreaction is used to perform a SERS-based immunosensor. Direct SERS detection of CA-125 is analyzed in the comprehensive review article by Geka et al., which describes two approaches. The first approach involves mixing Ag nanoparticles with human samples and placing them on aluminum foil slides. The second approach focuses on the immobilization of antibodies against CA-125 with Ag nanoparticles to observe changes in the Raman spectrum. Furthermore, the human epididymis protein 4 (HE4), a new ovarian cancer biomarker, was identified with SERS. Geka et al. examined a report that added 4-mercaptobenzoic acid (4-MBA) and HE4, modifying Au nanoparticles. Another approach involved the interaction of the anti-HE4 antibody with a single crystalline Au nanoplate coupled with thiol-modified protein, assessed using the Raman reporter malachite green isothiocyanate. The detection of various ovarian cancer biomarkers highlights the versatility of SERS-based immunosensors, as well as their low cost and high sensitivity, which have significantly contributed to their success [89].

In 2023, Lan et al. developed a newly performed Surface-Enhanced Raman Scattering (SERS) method targeting cyclophilin A (CYPA), an early ovarian cancer biomarker. The essential material, a yolk shell nanostructure, was prepared through a balanced ratio of HAuCl_4_ and AgNO_3_, enabling it to possess excellent SERS properties and photothermal activity. For active SERS applications, nanomaterials such as Au-Ag nanoalloys or Au@Ag nanohybrids encapsulated with SiO_2_ are utilized. Photothermal therapy (PTT) is integrated with SERS, employing the Au@Ag nanohybrids for their ability to convert light energy to heat at the tumor site, enabling the simultaneous detection and treatment of ovarian cancer. To initiate this process, CV acting as a Raman tag molecule must be absorbed into the surface of SiO_2_-encapsulated Au star@AgAu yolk shell nanostructure (Au@AgAu YSNS). Subsequently, coupling the antibody against the CYPA biomarker and the color emission of the substrate Tetramethylbenzidine (TMB) permits ovarian cancer detection at low concentrations due to high specificity. The Raman signal intensity increases with an increased ratio of Ag: Au and CYPA concentration, indicating that Au@AgAu YSNS with SERS promotes nano-theranostics. The sensor showed a higher sensitivity to detecting CYPA as low as 7.7 × 10^−10^ μg/mL [90].

**Figure 6:**
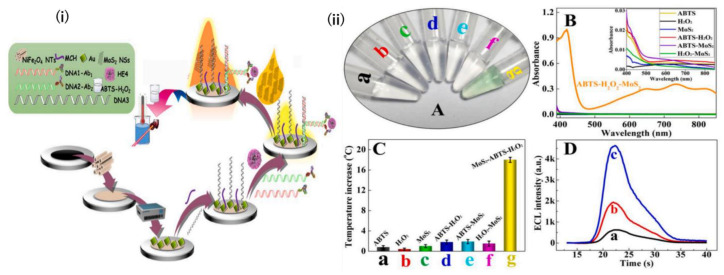
(**i**) Schematic illustration of the proximity hybridization-based multiple-stimuli-responsive immuno-sensing platform. (**ii**) (**A**) Photographs and (**B**) UV–vis absorption spectra of different components in MoS_2_ NSs-mediated ABTS-H_2_O_2_ colorimetric system. (**C**) Temperature changes in the corresponding reaction solutions (0.2 mL) after irradiating by an 808 nm laser (2.5 W cm^−2^) for 30 s. (**D**) ECL responses of (a) GCE and (b) GCE/MoS2 NSs without and (c) GCE/MoS2 NSs with 808 nm laser (2.5 W cm^−2^) irradiation for 40 s in 0.1 M PBS4 (0.1 M Na_2_HPO_4_, 0.1 M NaH_2_PO_4_, pH 8.0) containing 10^−5^ M luminol. a–f in (**A**,**C**) correspond with each other. This image was adapted from Zhang et al., 2020, with copyright permission [91].

A major advancement is the development of a split-type, multiple-stimuli-responsive biosensor that combines various optical biosensing techniques into one device by Zhang et al. The integration of electrochemiluminescence, which does not require an external light source, thereby reducing background interference, and colorimetry for simplicity and visual analysis, with photothermal sensing, makes it faster and more effective in measuring multiple signals. The key material to enhance the sensor is MoS_2_ nanosheets (MoS_2_ NSs), a graphene-like nanomaterial with good electrocatalytic properties and a photothermal effect. To apply this biosensor, NiFe_2_O_4_ nanotubes (NTs) were dipped in the matrix with electrodeposits of Au nanoparticles (ed-Au NPs) and captured thiolated DNA (DNA3) in an Au-S bond modified electrode. Subsequently, to perform the immunological reaction, different concentrations of the HE4 biomarker were added to the electrode. The colorimetric system and photothermal sensing take place when the ss-DNA is absorbed into the electrode, as increasing concentrations of HE4 biomarkers in a sample increase the hybridization of dsDNA (Figure 6) [91]. This causes less MoS_2_ NSs absorption and more residual MoS_2_ NSs to remain in the solution. The colorimetric system is employed to measure its performance, wherein a green color is produced from the oxidation of MoS_2_ NSs with colorless ABTS in the presence of H_2_O_2_. Zhang et al.’s innovation of a reproducible, highly sensitive, and economical product is pivotal in the progression of ovarian cancer diagnosis, particularly in the context of developing point-of-care diagnosis.

Numerous studies have emerged focusing on the detection of microRNAs (miRNAs) for diagnosing ovarian cancer, owing to their stability and presence in body fluids, which allows for their easy non-invasive extraction compared to other biomarkers. Accordingly, Ivanov et al. unveiled a silicon-on-insulator nanowire biosensor (SOI-NW biosensor) designed for miRNA detection. The silicon-on-insulator (SOI) structures are constructed using the CMOS-compatible top–down approach to produce the SOI-NW sensor chip. To operate, the oDNA probes carrying sequences complementary to miRNA will be covalently immobilized to sensitize the surface of the silicon nanowire with the activated DTSSP (3,3′-dithiobis(sulfosuccinimidyl propionate)) cross-linker, enabling the detection of the targeted biomarker. Several modifications in this design make it distinctive. For example, the platinum rod enhances the stability of the SOI-NW sensor chip and enables simultaneous detection of up to 10 wires. These signals are then converted into a graphical form using specialized software [92]. The reduced time and resources required for miRNA detection among ovarian cancer candidates, with high specificity to tissue type, highlight the advantages of incorporating miRNA in future biosensors. Using silicon-on-insulator nanowire biosensors that are label-free without amplification reactions, and enabling instantaneous identification, underscores Ivanov et al.’s significant contribution to advancing ovarian cancer diagnosis, and the LOD of this method was 1.1 × 10^−16^ M. Their discussion of its potential use as a routine bioassay for early screening further emphasizes the importance of their work.

### 2.2. Electrochemical Biosensors

Detection of ovarian cancer in its early stages using markers like CA-125 is crucial and can be achieved by various methods; one of them is through electrochemical sensing [93]. Although it is difficult to diagnose OC early [94], detecting biomarkers using electrochemical (EC) methods has proven to be fast, sensitive, easily miniaturized, and appropriate for point-of-care services [95]. An EC immunosensor, a type of EC biosensor, depends on the biorecognition reaction occurring when an antigen–antibody complex forms and the subsequent electrical output. EC biosensors implement a three-electrode configuration system, constructed using cheap materials and simple electronics, that obtains its flexibility and portability from a small amount of electrolytes. All these features offer several advantages, including ease of use, low cost, and multi-analyte testing capability to these sensors, which are usually used as non-invasive, point-of-care tools. It is essential to have a sensitive and specific platform for biosensing to ensure excellent biocompatibility, high electrical conductivity, and the materials’ active surface area. Amperometric advantages of EC biosensors include low cost, high sensitivity, simple operation, and portability, while some disadvantages are time consuming, sensitivity to the surrounding environment, and signal reduction. Additional potentiometric pros are rapid response, small size, high selectivity, lack of sample treatment, while cons are sensitivity to temperature and pH, and limited applications. Impedimetric advantages resemble the amperometric, while additional disadvantages are complex construction and expensive labeling markers. These biosensors are better for achieving overall estimation when an exact answer with high selectivity is not needed.

Electrochemical immunosensors for the detection of ovarian cancer biomarkers mainly rely on the formation of antigen–antibody complexes termed biorecognition events [96]. These sensors have been categorized into label-free and sandwich-type EC immunosensors. The former does not utilize labels but rather directly detects the binding of the target analyte with the biorecognition element, eliminating issues associated with labels, like time consumption and binding site alteration that affect sensitivity. Label-free and sandwich-type EC biosensors have been developed for detecting Carcinoembryonic Antigen (CEA), Cancer Antigen 19-9 (CA 19-9), Alpha-fetoprotein (AFP), p53, Cancer Antigen 15-3 (CA 15-3), CA-125, HER2, and (HE4), all of which have multiple modalities. Furthermore, multiplex EC immunosensors are a relatively new formulation that involves the simultaneous detection of more than one marker, decreasing the time spent for running tests. However, there is a limited number of studies on multiplex EC. Alongside the conventional markers for ovarian cancer detection (CA-125 and HE4), other predictive biomarkers have been used, including circulating tumor DNA (ctDNA), DNA methylation, and tumor-specific autoantibodies [53]. Unfortunately, background noise due to side reactions could affect the sensitivity of EC biosensors [96]. In order to tackle this issue, light energy has been added to the system, creating a photoelectrochemical sensing platform currently known as PEC (photoelectrochemical sensing), a combination of EC detection and photo irradiation, similar to optical methods. It is important to note that gold nanoparticles (AuNPs) are widely used in electrochemical biosensing.

Extracellular vesicles are defined as lipid-bilayer-encapsulated particles secreted by cells into the extracellular space, shuttling various molecules, and exhibiting an intricate biological character [97]. The molecules shuttled and, subsequently, the biomarkers detected are divided into proteins, lipids, and nucleic acids. Examples of EVs-related proteins in the context of ovarian cancer tumor biomarker detection include CD9, MUC1 (detected by aptamer-conjugated spiky Au@Fe_3_O_4_), and simultaneous detection of various markers like CD63, EpCAM, CD24, and CA-125 to avoid false positives. As for EVs-related nucleic acids, miRNAs, mRNAs, and circular RNAs (circRNAs) are included, with the former being the most studied and portraying significant abnormalities in cancer. Electrochemical sensing methods have been used to detect small extracellular vesicles, specifically, miRNAs, for the diagnosis and prognosis of ovarian cancer [54]. Examples of these methods include nanopore sequencing and microfluidic chips, offering not only excellent sensitivity and specificity in detection but also real-time monitoring of disease progression and treatment outcomes. Exosomes, a type of cell-derived, extracellular, nanoscale vesicles that transport various elements like DNA, RNA, and proteins have a crucial role in regulating multiple cellular processes including tumor cell proliferation and differentiation [94,98]. Hence, by identifying cancer-derived exosomes, a novel category of non-invasive biomarkers can be used for early detection [94]. Deng et al. have designed an entropy-driven autocatalytic DNA circuit (EADC), a sensitive and accurate electrochemical biosensor for the detection of exosomes derived from ovarian cancer cells (2024). EADC is a nucleic acid self-assembly system that mainly involves DNA probes, used for the recognition of target exosomes, as well as DNAzymes for cleavage reactions, and a DNA circuit for autocatalytic function. The combination of these elements offers a sensitive and remarkable low detection limit of 30 particles/μL. This was evident when comparing control and patient samples, deeming this biosensor as a promising tool for clinical diagnosis. Furthermore, Ge et al. prepared a double-hook type aptamer EC sensor using metal–organic frameworks [94]. Through EIS, a good linear relationship within the concentration range of 31–3.1 × 10^6^ particles per microliter and a low LOD (12 particles per microliter) was achieved. This platform is distinguished for its ability not only to differentiate between healthy people and high-grade serous ovarian cancer (HGSOC) patients, but also to discern the latter from non-high-grade serous OC (non-HGSOC) patients.

#### 2.2.1. Label-Free and Sandwich-Type EC Biosensors

Detection of OC by the use of label-free immunosensors is essential to improve diagnosis and protect women from this life-threatening disease [99]. An ultrasensitive and label-free electrochemical immunosensor was devised by Mu et al. for the detection of CA-125 [100]. Copper–cobalt oxide nanosheets and gold nanoparticles (CuCo-ONSs@AuNPs) were incorporated into the sensor’s structure. The CuCo-ONSs functions to provide strong output signals as well as adhesion sites for the AuNPs owing to its two-dimensional architecture. Then, AuNPs having multiple active sites that facilitate the immobilization of biomolecules, and the antigen–antibody complex formation produces an electrochemical response. This EC immunosensor obtained a linear detection range from 1 × 10^−7^ U/mL to 1 × 10^−3^ U/mL and a detection limit of 3.9 × 10^−8^ U/mL (S/N = 3). Moreover, implementing CuCo-ONSs@AuNPs has eliminated the need for pre-activation and additional cross-linkers. Another label-free CA-125 immunosensor was made using gold nanoparticles and poly toluidine blue (PTB) in deep eutectic solvent placed on screen-printed carbon electrodes, providing reliable, feasible, and environmentally friendly clinical detection [101]. After evaluation of the immunosensor through various modalities and the analysis of CA-125 by EIS and this immunosensor, it was concluded that CA-125 levels can be obtained quickly, with adequate repeatability, within a low limit of detection (1.20 pg/mL) and in the linear range of 5–100 pg/mL. Tests were run using human blood serum, which yielded good results. Therefore, this disposable, label-free, and impedimetric immunosensor is appropriate for conducting CA-125 detection tests at point-of-care. So, the development of disposable, point-of-care immunosensing techniques has greatly benefitted the monitoring and treatment of patients [102].

Interestingly, a study used a hierarchical microporous carbon material fabricated from waste coffee grounds (WCGs) to devise a simple label-free EC immunosensor [103]. WCGs were pyrolyzed with potassium hydroxide and used to modify a screen-printed electrode, which was then decorated with AuNPs to capture the specific antibody. Cyclic voltammetry (CV) and electrochemical impedance spectroscopy (EIS) were used to characterize the modification and immobilization process. Results yielded an effective dynamic range of 0.5 to 50.0 U/mL, a 0.9995 correlation coefficient, and an LOD of 0.4 U/mL. Human serum analysis depicted similar results, confirming the accuracy and precision of this dual immunosensor. Additionally, a few studies used glassy carbon electrodes (GCEs) for the building of their biosensors. An electrochemical CA-125 aptasensor was synthesized using graphitic carbon nitrides, molybdenum disulfide, and magnetic nanoparticles (g-C_3_N_4_/MoS_2_/Fe_3_O_4_) immobilized on a GCE [104]. A significant feature of this aptasensor is its inclusion of both label-free and labeled forms of detection, catalyzed by ferrocyanide and methylene blue, respectively. Both obtained a low LOD of 0.215 U/mL and 0.202 U/mL, respectively, and a broad detection range (2 to 10 U/mL). A comparison between serum samples of patients and normal people demonstrates this sensor’s significant application, and its high affinity and stability in terms of CA-125 detection supports that even more. In a different study, to set up a carbon nanomaterial/gold nanocomposite, multi-wall carbon nanotubes (MWCNTs), vapor-grown carbon fiber (VGCF), graphite KS4, and carbon black super P (SP) were treated with acids [99]. Next, this AuNPs@carbon nanocomposite was electrochemically deposited on a glassy carbon electrode for 50 cycles, serving as a substrate for manufacturing this CA-125 detecting immunosensor. The AuNPs@MWCNTs-based sensor displayed the highest CA-125 sensitivity (0.001 µg/mL) by square wave voltammetry (SWV). MWCNTs’ surface area and increased conductivity aided the AuNPs’ immobilization, and its carboxylic functional groups provided great support for the sensor’s fabrication after treatment with acid. A layer-by-layer (LBL) assembly of the AuNPs@carbon nanomaterials EC biosensor is the main target of this novel method, as it can aid in point-of-care clinical diagnosis while maintaining a low cost.

Furthermore, researchers have developed dual-EC immunosensing techniques. A label-free CA-125 and HE4 sensor has been developed using disposable screen-printed carbon electrodes modified with reduced graphene oxide, polythionine, and AuNPs for timely and practical detection of these two markers’ levels [102]. EC detection of antigens within four different linear ranges (1–100 pg/mL, 0.01–10 ng/mL, 10–50 ng/mL, and 50–500 ng/mL) was performed using EIS, square wave voltammetry, and differential pulse voltammetry. Results yielded a low LOD, high sensitivity, and limit of quantification (LOQ) for each linear range with a 0.99 correlation coefficient. A 60-day period was determined for the immunosensors’ application stability, along with a storage stability of 16 weeks. High selectivity was evident in nine various antigen mixtures. Point-of-care testing of blood serum samples at the pg/mL concentration using a handheld EC reader demonstrated high recoveries within 20–30 s. Therefore, label-free and disposable immunosensors are suitable point-of-care evaluation methods, user-friendly, and show high sensitivity, selectivity, and repeatability when it comes to the detection of CA-125 and HE4. Another label-free immunosensor was put together for the simultaneous detection of CA-125 and HE4 [105]. In its structure, disposable dual screen-printed carbon electrodes were modified with AuNPS, reduced graphene oxide, and polythionine, providing sensitivity and rapid response time. DPV and SWV were the methods utilized to detect these two antigens in two different linear ranges (1–100 pg/mL and 1–50 ng/mL), and both achieved high sensitivity and low LOD and LOQ, with a correlation coefficient above 0.99. Furthermore, this dual CA-125–HE4 immunosensor has an application stability of 60 days, 16 weeks storage stability, high selectivity in eight different antigen mixtures, and a nine-cycle reusability. Therefore, this form of testing can be used at point-of-care for quick and practical detection of both CA-125 and HE4 with high sensitivity, selectivity, and repeatability.

In addition to that, a dual-signal ECL immunosensor was manufactured [106]. However, it is a sandwich-type immunosensor and not a label-free one. Anodic signals were strongly seen with Eu metal–organic framework-loaded isoluminol-Au nanoparticles (Eu MOF@Isolu-Au NPs) through a synergistic interaction, while cathodic signals were obtained from the composite of carboxyl-functionalized CdS quantum dots and N-doped porous carbon-anchored Cu single-atom catalyst, catalyzing H_2_O_2_ as a co-reactant for the production of ^•^OH and O_2_^•−^ in large amounts. This promotes a significant increase in this ECL sensor’s signaling and stability. Moreover, this sandwich-type immunosensor was established based on the enhancement strategy through the combination of antigen–antibody specific recognition and the magnetic separation technique. This immunosensor had high sensitivity; great selectivity; stability; practicality; a wide linear response range of 0.005∼500 ng/mL; and low detection limits of 0.37 and 1.58 pg/mL for CA-125 and HE4, respectively, hence building a blueprint for in-depth design and single-atom catalysis application in the field of ECL biosensors. A further study prepared a sandwich-type EC immunosensor only for CA-125 detection using conductive composite materials of carbon ink/carbon dot/zinc oxide (C-ink/CD/ZnO) and ITO substrate to enhance the interaction of antibodies (Abs) [107]. The ZnO particles supported the electrochemical performance of the assay, while also functioning as a labeling signal molecule. In addition, the nanocomposite of silver@polypyrrole (Ag@PPy) served as a potential redox mediator, and labeling it yielded more accurate results than the label-free formulation. A linear range of 1 ag/mL to 100 ng/mL and a low LOD (0.1 fg/mL) comprised this novel immunosensor’s characteristics, making it an efficient tool for clinical diagnosis.

Nonetheless, while EC biosensors have proved to be quite valuable, a challenge faced in the development of this type of sensor is non-specific adsorption (NSA), which involves the fouling of non-target molecules in the blood on the recognition surface of the tool [95]. Ahmadi et al. have assembled an affinity-based EC biosensor that incorporates a novel technique of using silane-based interfacial chemistry to overcome NSA and medical-grade stainless steel electrodes to reduce costs and improve sensitivity, all for the detection of lysophosphatidic acid (LPA). LPA was found to be increased in 90% of stage one ovarian cancer patients and is further elevated with the progression of the disease. By utilizing the affinity-based gelsolin–actin system, a biorecognition surface for detecting LPA, it has been concluded that this label-free biosensor has a detection limit of 0.7 µM and a linear range of 0.01–10 µM in goat serum as a proof-of-concept for the prompt diagnosis of ovarian cancer.

#### 2.2.2. Electrochemical Aptasensors

Gold nanostructures (GNs) have also been used in the development of an electrochemical aptasensor for detecting CA-125 [108]. After depositing GNs on fluorine-doped tin oxide electrodes, these modified electrodes’ properties were evaluated via cyclic voltammetry, electrochemical impedance spectroscopy, field emission scanning electron microscopy, and X-ray diffraction. The electrode of the best quality was used for the assembly of a CA-125 tumor marker aptasensor. Uniform deposition of GNs and an increased electroactive surface area signified the best electrode, which showed a detection limit of 2.6 U/mL and a linear range of 10 to 800 U/mL, eliminating the need for costly antibodies to detect ovarian cancer markers. Moreover, Hu et al. introduced a one-step electrodeposition process to develop a novel AuNFs@MoS_2_ nanohybrid for the aptamer-based electrochemical detection of CA-125 [109]. The gold nanoflowers (AuNFs) provided the sensing interface with a large specific surface area, the molybdenum disulfide (MoS_2_) served as the stable layered substrate, and the modification step increased the performance of the electrode while also offering multiple sulfhydryl binding sites. Gold sulfur bonds allowed the fixation of CA-125 aptamer. In order to reduce non-specific adsorption (NSA), 6-Mercapto-1-hexanol (MCH) was used. The final analysis of this biosensor by differential pulse voltammetry (DPV) not only showed a detection range of 0.0001 U/mL to 500 U/mL, but also exhibited stability, reproducibility, stability, and clinical feasibility.

Aptasensor-based EC biosensors can be classified into amperometric/voltametric, impedimetric, photoelectrochemical (PEC), and electrochemiluminescence (ECL) methods [110]. Application of electrochemiluminescence in an aptasensor using tetrahedral DNA nanostructure (TDN) enhanced toehold-mediated strand displacement (TMSD) was devised [111]. AuNPs and Ru(bpy)_3_^2+^-modified ZIF-MOF were coupled to TMSD, used for initial ECL signaling, and hybridized with S5 and ferrocene-labeled DNA probe S6. Ru(bpy)_3_^2+^ emitted an ECL signal that was absorbed by S6, putting the sensor in a “signal-off” state. Through the binding of CA-125, S7, DNA initiator, and S8, the helper DNA was released, inducing the detachment of S6 and, ultimately, the ECL signal retrieval. This puts the aptasensor in a “signal-on” state. To prepare for another cycle of TMSD, S7 is recycled. The presented ECL aptasensor achieved a detection limit of 6 × 10^−3^ pg/mL and a high performance in human serum samples, enhancing its potential for clinical analysis.

Two-dimensional MXene is currently a new focus in the field of biosensing, particularly for its optic and electrical properties [112]. However, due to its low stability, MXene–Metal interactions are used to increase MXene’s durability. After testing multiple MXene/Ag nanocomposites, such as Ti_3_C_2_/Ag, Nb_2_C/Ag, and V_2_C/Ag, it was evident that V_2_C MXene had the strongest self-reducing ability, and incorporating it into an electrochemiluminescence–photothermal immunosensor was beneficial for the detection of lipolysis-stimulated lipoprotein receptor. Another novel application for the detection of CA-125 using MXenes is a sandwich-like electrochemical immunosensor (STEM) involving a primary (PAb) and secondary (SAb) antibody [113]. In order to immobilize these two antibody groups, Ti_3_C_2_T_x_ MXenes (Ti_3_C_2_T_x_NR) and UIO-66-NH_2_ MOFs structure were used, respectively, with the latter also serving to immobilize the electroactive toluidine blue (Tb) probe. The Ti_3_C_2_T_x_NR nanohybrid provided a large surface area and proper conductivity as a carrier, while UIO-66-NH_2_ offered a proper platform to house Sab, and Tb molecules of peak currents increased in proportion to CA-125 levels. This STEM structure’s very low detection limit (0.05 U/mL) and wide linear range (0.2–150.0 U/mL) may have clinical implications in the future.

Finally, Maghiani et al. explored the application of NiFe_2_O_4_ magnetic nanoparticles in electrochemical sensing platforms [114]. Structural, magnetic, and morphological properties, along with cost-effectiveness, add to this material’s benefits. After functionalization with cystamine, glutaraldehyde, and PVDF, CA-125 antibody was incorporated and successfully immobilized. To be utilized as a point-of-care device, this immunosensor was assembled on a printed circuit board. Results showed excellent specificity, selectivity, stability, and reproducibility. Therefore, it is clear that the field of electrochemical biosensors, while intricate, is massively impacting the clinical diagnosis and treatment of ovarian cancer by permitting the early detection of certain tumor markers with great sensitivity and repeatability. Moreover, their current design allows physicians ease-of-use, portability, and disposability. Also, EC sensing is an ever-evolving area, promising a great future for battling ovarian cancer.

## 3. Mass-Based Biosensors

Mass-based biosensors are analytical devices that detect target biomolecules by measuring changes in mass. They are also called piezoelectric biosensors, which operate based on piezoelectric crystal resonance frequency detection secondary to changes in the crystal mass. They are further divided into two types called the quartz crystal microbalance (QCM) and the surface acoustic wave device (SAW) [115].

QCM uses a quartz disc with dual-sided electrodes, which, when placed in an electric field, causes a mechanical stress on the crystal surface and makes it oscillate proportional to its mass per unit area. When biomolecules bind to this crystal, they cause a structural change at the crystal surface and reduce its oscillations. QCM biosensors capitalize on this property, as mass is a property of all molecules, and any target analyte can bind and cause a change in crystal resonance frequency. This makes QCM able to identify any type of molecule. However, any mass can alter frequency, so to improve its specificity, the crystal is embedded with receptors that bind only to target molecules. Furthermore, the test is highly sensitive, as QCM can detect even the smallest of changes on the crystal surface mass, and results are rapid (<1 h), as there is no need for additional labeling of the molecules. QCM receptors can range from being antibodies to nucleic acids [116]. The literature describes the use of QCM in detecting ovarian cancer cell antibody NuTu-19. The crystal surface was functionalized using NuTu-19 cells, and the serum samples were introduced. A change in mass reflected by the antibodies being adsorbed onto the crystal surface was measured by monitoring the resonance frequency of the crystals, which were directly proportional to the antibody mass [117]. Another study looked at QCM detection of mesothelin, which is a protein that can be found on ovarian cancer cells. Mesothelin-specific antibody was used for functionalization in this case, and the QCM biosensor was able to detect mesothelin from 100 pg/mL to 50 ng/mL [118].

The SAW device measures analytes by detecting changes in surface sound waves due to mass loading from biomolecular interaction. Its surface is functionalized with a coating specific to the analyte. One study looked at Bcl-2 as a biomarker for ovarian cancer diagnosis using SAW with specific functionalization to capture this protein and the response measured as a frequency shift in the wave. This study demonstrated the high sensitivity and specificity of SAW, as it was able detect Bcl-2 from a protein mixture [119]. SAW devices can be influenced by non-specific binding of other serum proteins that can reduce the sensitivity of the device; hence, a new development has introduced a hexagonal transducer. This sends waves with different features in different directions, thus making the device useful for multiplexing and reproducibility of the experiment [120].

Ultimately, the nanomechanical motions are picked up by a reader, such as using optical beam deflection. It is advantageous due to fast results, efficient sample preparation, and lack of need for labeling. A downside of this system is the heat energy produced that can limit the level detectable by the biosensor [121]. Cantilevers have been claimed to be useful for detecting tumor biomarkers [61]. The microcantilever has been shown to be efficacious in detecting MUC1, which is overexpressed in ovarian cancer. This sensor measured changes in surface stress and was able to detect levels as low as 0.9 nM. In the experiment, the microcantilever was fixed to a holder, and the free end was placed near a laser beam, whose deflections were recorded through a camera. A downward bending of the microcantilevers indicated compressive stress, while an upward bending indicated tensile stress [122].

## 4. Microfluid-Based Sensors

Microfluidics is a modern technology for processing and manipulating the fluids in the microchannels, a microminiaturized device built with chambers and tunnels through a small volume (in femtoliter, f/L) of fluid flow. The behavior of the fluid is different in a microfluidic channel compared to the bulk flow. This unique nature of the behaviors is used for novel scientific experiments and new inventions. The microfluidic chip, or Lab-on-a chip, exhibits a promising application in the field of cancer therapy. It has potential applications for developing preclinical models for cancer, cancer biomarkers for diagnosis, anti-cancer drug screening, analysis of tumor heterogeneity, and nanodrug design and production [123]. Zhao et al. developed a microfluidic method for the detection of tumor-derived circulating exosomes. They used multiplex detection using immunomagnetic beads. The continuous-flow design was used for the quantitative detection of exosomes from blood plasma. This microfluidic approach was employed for the multiplexed detection of blood-based ovarian cancer from three different exosomal tumor markers, such as CA-125, EpCAM, and CD24. The analysis report was comparable with the standard Bradford assay. This method can quantitively detect exosomes as low as 7.5 × 10^5^ particles/mL; this is a 1000-fold lower LOD compared to Western blotting [124].

## 5. Paper-Based Lateral Flow Assay

Cancer detection depends on an equipment’s sensitivity to identify low concentrations of biomarkers, and technology available for that task is both expensive and requires trained personnel. Hence, a paper-based detection option was developed, which includes the lateral flow assays (LFAs) [125].

LFAs are dubbed lateral due to their direction of sample flow secondary to capillary action [126]. The assay is arranged as four consecutive paper pads made of cellulose or porous nitrocellulose. The first one is the sample pad, which collects the sample and ensures homogenous delivery to the conjugate release pad, where the fluorescent particles of gold or latex-conjugated antibodies are present. The biomolecule–antibody conjugate then encounters the pad that contains the test and control strip. These strips contain immobile antibodies that then bind to the conjugate and change color, showing the presence of the biomolecule. The control line changes color to further authenticate the fluid flow. The last absorbent pad mops up any excess fluid and prevents back flow. The results are then detected by a visual confirmation or put through an optical reader [127]. They are of two types: direct and competitive. Large analytes are handled by the direct type and smaller molecules by the competitive type [126]. A further classification can be based on the elements involved in the assay: ones that use antibodies are called lateral flow immunoassays (LFIAs), and those that use nucleic acids are called nucleic acid lateral flow assays (NALFs) [127]. NALF has been described in a study to detect nucleic acids using a single-stranded DNA aptamer that specifically binds to CA-125 at varying concentrations to detect ovarian cancer [128].

LFAs are widely recognized for their rapid turnaround time, delivering results in just 5–30 min. They are cost-effective and can be used with minimal training. Additionally, LFAs are favorable in resource-limited settings due to their long shelf life and lack of refrigeration requirements. However, they have some limitations, including potentially lower sensitivity and specificity compared to historically used methods, and they often provide qualitative results rather than precise measurements. Furthermore, while they offer multiplexing capabilities, the number of analytes detectable in a single test is limited [127].

A recognized LFA kit that is commercially available for ovarian cancer detection is the Quicking Biotech Co. Ltd., which uses only 100 μL of serum to detect CA-125 within 5–10 min, and it has a sensitivity of 40 U/mL [126].

LFAs can be designed for the detection of multiple analytes in a single test and have been used in point-of-care diagnostics, such as in cancer detection. Common ovarian cancer biomarkers detected using LFA include CA-125 and HE4. One such study utilized paper-based LFA to detect ovarian cancer recurrence from the HE4/creatinine ratio in urine using cell phones as the reader. Creatinine was used to add a layer of standardization, as HE4 levels can fluctuate with urine volume. Monoclonal mouse anti-human epididymis protein 4 was used as the antibody, and BioReady150 nm gold nanoshells was the reporter reagent. Sometimes, HE4 and creatinine levels may exceed lateral flow test limits, requiring urine dilution, which can introduce errors if done by patients. Hence, using a precise pipette with pre-measured deionized water was suggested to minimize these errors. The study highlighted that the ease of use and less invasive nature of the test makes it more attractive to patients. Furthermore, it recommends measuring HE4 levels in urine at ovarian cancer diagnosis and post-treatment to provide a biomarker benchmark. Monitoring HE4/CRE levels from this point onwards can then guide the need for further testing [129].

Recent studies have also looked at improving the readability of optical markers on LFIAs by using hydrophobic gold nanoparticles at high density implanted onto Magnesium and Ferric cations with layered double hydroxides. This improved the optical signal intensity of the carrier and the overall sensitivity of the test [130]. Another study overrides the current limitations of colloidal gold-based LFAs with upconverting nanoparticles (UCNPs). These are lanthanide-doped nanoparticles with fluorescent properties that can be detected by an infrared laser light, thus replacing subjective visual detection with a quantitative approach that has better sensitivity for point-of-care diagnosis [131]. UCNPs have also been used in multiplexed LFAs, which can detect multiple biomarkers from a single sample. It has improved diagnostics, as individual cancer biomarkers are often non-specific. This was achieved by utilizing dual-labeled UCNPs doped with erbium and thulium, which allowed detection of different antigens in one test line [132]. The fluorescent LFA technology also heavily depends on the quality of the fluorescence signals produced to detect low concentrations of analytes. Hence, Wang et al. used particles called dendritic mesoporous silica nanoparticles (DMSNs) to improve the sensitivity of FLFIA. DMSNs were larger in size, allowing them to carry more fluorescent molecules, and were more stable, ensuring consistent fluorescence signals [133].

## 6. Field Effect Transistor-Based Sensors

A field effect transistor is an electrical biosensor and utilizes the change in current flow to detect the presence of target molecules. Electrical biosensors have a receptor and transducer. The former identifies the biomolecule by forming a chemical bond with it. The latter converts this binding into a detectable electrical input. FET devices are made up of a channel, electrode, and base substrate. The electrodes are further divided into three types: source, drain, and gate. The source and drain are connected using a semiconducting material, and the gate controls current flow between them by establishing a channel. The gate can be a top gate or a back gate. The base substrate in FET devices is non-conducting. The generated electrical signals are then measured using a semiconductor parameter analyzer by a voltage difference between the source and gate. Thus, it can be understood that these values are influenced by several factors, like gating effects, molecule absorption, and gate capacitance [134]. FETs based on a micro/nanoelectromechanical system work by converting a chemical reaction into an electrical signal that is proportionate to the analyte concentration. The receptor in these FETs can be an antibody, enzyme, DNA, or single-stranded nucleic acid [135].

FETs setup is often used as a transducer and can be made of several semiconducting materials, such as metal–oxide semiconductors, organic semiconductors, or carbon nanomaterials. Carbon has been a preferred choice due to the slowness of the other two materials [134]. The source and drain electrodes are made of materials that have low resistance and good conductivity, such as gold with chromium or titanium adhesion [135]. FET does not rely on signal generators and can respond to any target through its gating mechanisms [134]. FET methods are favored due to their quick results turnaround time and compact technology that renders it portable [136]. They eliminate the need for fluorescent tags that complicate optical sensing methods. FET is also highly sensitive, as it can amplify electrical signals and detect even small changes in charge easily [137]. In recent times, it has been gaining ground due to the potential of integrating it with computer circuitry, the ability to use it on-site, and production adaptability [138]. In contrast, some limitations have also been identified with FETs with regards to uneven nanocarbon deposition, difficulty of preparing the nanocarbons, and random binding [134].

One study describes the use of FET for the detection of CA-125. The first FET sensor for CA-125 used a multi-walled carbon nanotube–reduced graphene oxide composite on a polymethyl methacrylate (PMMA) substrate with electrodes of gold and platinum. The tubes are first treated to create reactive sites and then coated with single-stranded DNA that specifically targets CA-125. This sensor exhibited good sensitivity and was able to detect concentrations as low as 5.0 × 10^−10^ U/mL [135,139]. Recent advancements in FET have brought new 2D materials like graphene, transition-metal dichalcogenides, and black phosphorus to light, with greater surface area compared to volume. This is aimed at improving the test’s sensitivity. Furthermore, other studies are looking to eliminate additional amplification steps for DNA/RNA during analysis. This is aimed at expediting the results. The third emerging trend is integrating non-silicon FETs with complementary metal–oxide semiconductor technology to support analysis directly on the chip without needing external devices. This would in turn allow real-time data collection and enable point-of-care diagnostics. Lastly, silicon-based open-gate junction FETs were used for the first time to sense DNA directly, eliminating the need for traditional reference electrodes [137].

Another innovation in FET biosensors was the introduction of an indium–selenide channel (InSe FET), which showed substantive electrical stability in a liquid medium. This was possible due to a process called “passivation”, which involves coating the channel surface with a non-reactive protective layer. The InSe FET was able to quantitatively detect CA-125 levels as low as 0.01 U/mL in under 20 min, making it suitable for early diagnosis of cancer [140]. Due to the short channel length that limits the sensitivity and the high subthreshold swing (SS) needed for the off-to-on state of FETs, further research developed a tunnel field effect transistor (TEF). TEFs use a mechanism called quantum tunneling to lower the SS to below 60 mV/dec, which in turn allows electrons to pass through a barrier that traditional transistors cannot overcome. This allows for a faster off-to-on switching speed and lower energy consumption [141]. However, TFETs posed a further challenge of recognizing well-defined transitions, which creates a junction for tunneling. Thereby, the next wave of research brought the junctionless tunnel field effect transistor (JLTFET), which combined junctionless technology with tunneling. This has shown to decrease what is called a dopant fluctuation seen in TFETs by performing a uniform doping in JLFET. This produces superior results compared to FETs and TFETs, as it offers better biosensing, lower energy usage, and easier manufacturability [142].

## 7. Molecular Imprinting Polymers as Bioreceptors

**Figure 7:**
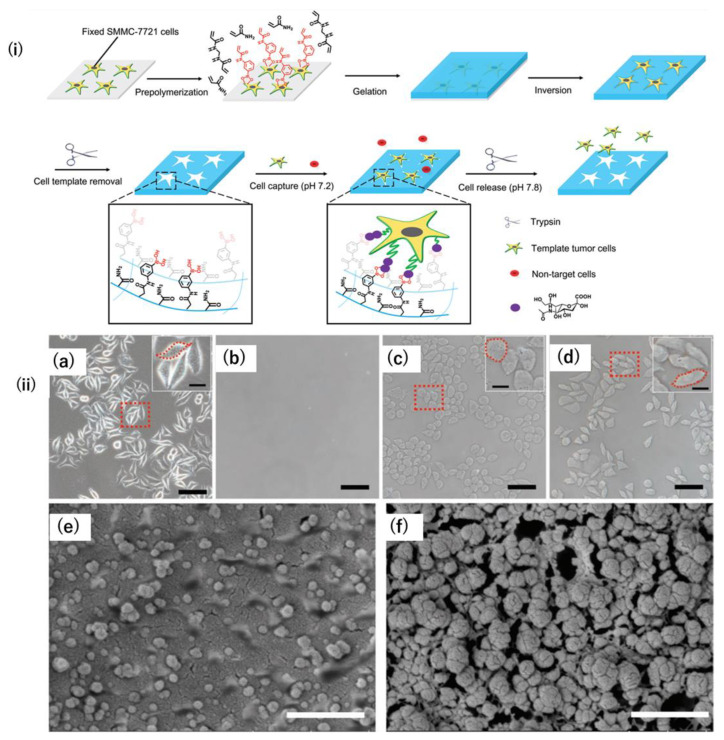
(**i**) Fabrication of the hydrogel with the synergistic effect of cell imprinting and boronate affinity (PBA-CIH) for the capture and release of SMMC-7721 cells. (**ii**) Microscopy images of the SMMC-7721 cells on (**a**) culture dishes and showing the surface morphology (**b**) NIH, (**c**) UCIH, and (**d**) CIH after removing the cell templates. The insets are zoomed in on the imprinted sites in the microscopy image. Cryo-SEM of the nanostructures inside the microstructures of (**e**) the UCIH and (**f**) the CIH. Scale bars (**a**,**d**) (**a**–**d** 100 µm) (**e**) represent 340 nm, and scale bar (**f**) represents 500 nm. The image is adapted from Liu et al., 2020, with copyright permission [147].

Molecular imprinting (MI) is a technique to create a target-specific receptor with high affinity from synthetic materials. Molecular imprinting (MI) technology is an emerging tool and low-cost biomolecule-based recognition receptor implemented in many sensor applications. MI has attracted widespread interest in the biomedical field due to its recognition specificity of targets, structure prediction, robustness, and simplicity [143,144]. The Molecular imprinted polymers (MIPs) are cross-linked polymers that are cross-linked in the presence of a template. The three-dimensional target-binding cavities are created within the polymeric matrix. The cavities are complementary to the size, shape, and fit functional group of the target molecules. The target molecule with the exact structure, shape, and orientation of the functional groups can only be allowed to occupy the cavity. This technology is often used for the development of biosensors in prognosis and diagnosis applications [145,146]. Figure 7 represents a cell-imprinted polymer hydrogel prepared from 3-(acrylamido) phenylboronic acid (3-AAPBA) functionalized for the selective capturing of tumor cells. Human hepatocarcinoma SMMC7721 cells were used as templates to produce the MIP. Finally, the cells are peeled off, and the resulting polymer matrix cavity is used for a tumor cell recognition receptor [147].

Liu et al. demonstrated an efficient capturing of circulating tumor cells (CTCs) from blood. They have fabricated an antibody-free hydrogel-based MIP for recognizing human hepatocarcinoma SMMC-7721 cells. A total of 4.5 cm^2^ MIP hydrogel was captured with an efficiency of 90.3 ± 1.4% (n = 3) from 1 × 10^5^ SMMC-7721 cells, and 99% of the cells were released without any loss in proliferation ability. The MIP matrix selectively captures the target cells in the presence of a high quantity of non-specific target cells (Figure 7) [147]. One of the important ovarian cancer markers, CA-125, level in serum was determined from a nanoscale surface plasmon resonance (SPR) biosensor using molecular imprinting technology. Freshly synthesized Poly(2-hydroxyethyl methacrylate-*N*-methacryloyl-(L)-tryptophan methyl ester) (p(HEMA-MATrp)) nanoparticles (NPs) were used for imprinting CA-125 and coated on the SPR gold chip. Non-imprinted p(HEMA-MATrp) nanoparticles were used as a control. The developed MIP-SPR biosensor was assessed using CA-125 glycoprotein in the dynamic range of 0.1–10 U/mL, with an 0.01 U/mL LOD. The selectivity and the real sample studies confirm the reliability of the CA-125 sensor from the serum [148].

Rebelo et al. developed MIP on a gold electrode surface for recognizing CA-12, an OC biomarker. CA-125-recognizing MIP was generated from the electropolymerization of pyrrole monomer on a gold electrode by cyclic voltammetry (CV). Square wave voltammetry (SWV) was used for testing the CA-125 detection analysis. The MIP-based electrochemical sensor selectively detects CA-125 targets in the concentration range of between 0.01 and 500 U/mL, with an LOD of 0.01 U/mL [149]. Another research team has developed a protein-imprinted polymer on a three-dimensional gold nanoelectrode ensemble (GNEE) for the CA-125 biomarker by cyclic voltammetry. A thin film of GNEE was the tool for capturing CA-125. Differential pulse voltammetry (DPV) and electrochemical impedance spectroscopy (EIS) were used for the detection of CA-125 in the detection range of 0.5–400 U/mL, with an LOD of 0.5 U/mL. Validation of this method was performed with a CA-125-spiked blood sample, and the non-specific biomolecules in the blood did not influence the sensitivity of the assay [150]. Büyüktiryaki et al. have used metal chelating monomer methacryloyl antipyrine europium (III) [(MAAP)2-Eu(III)] and methacryloyl antipyrine terbium (III) [(MAAP)2-Tb(III)] for MIP by metal chelating interactions. Phosphoserine (PS) was used as a template for CA-125 capture. The cavities in the PS-imprinted carbon nanotube (CNT) Fe_2_O_3_ nanoparticle selectively bind CA-125. The binding affinity was determined from Langmuir adsorption isotherms. The LOD of the sensor for PS and CA-125 was 0.177 nM and 0.49 U/mL, respectively [151].

Simultaneous detection of two different cancer biomarkers (CS125 and CA 15-3) has been reported using magnetic molecularly imprinted polymers (MMIPs). The MMIP was prepared from 4-carboxyphenylboronic acid as a functional monomer and tetraethyl orthosilicate (TEOS) as a cross-linker by the hydrothermal process. Biocompatible Ni NC- and Cd NC-capped bovine serum albumin (BSA) were used as fluorescence probes. The antibodies of CA-125 and CA 15–3 functioned as template molecules. A sandwich immunoassay was developed from the primary antibody–antigen fluorescently labeled secondary antibody. The targets CA-125 and CA15-3 were used in the rage of 0.0005–40/mL with an LOD of 50 μU/mL [70].

## 8. Discussion and Opportunities

Ovarian cancer remains a significant challenge due to its high mortality rate, primarily resulting from late-stage diagnoses. Early detection is crucial for improving patient survival; it enables more effective interventions at earlier cancer stages. However, traditional diagnostic methods, such as imaging and the CA-125 as biomarker, have limited sensitivity, particularly for early-stage ovarian cancer [152]. In the last decades, biosensors have emerged as a promising technology by offering a non-invasive, efficient, and potentially more accurate means of early detection and monitoring for ovarian cancers. Recent development in biosensors using nanomaterials has been summarized in the form of table in Table 1. Advances in biosensor technologies, particularly those utilizing nanomaterials and molecular recognition elements, have significantly enhanced the sensitivity and specificity of ovarian cancer detection. Nanomaterials, including gold nanoparticles and carbon nanotubes, offer large surface areas for biomarker capture, improving detection sensitivity [153]. These technologies, combined with techniques such as electrochemical, fluorescence-based, and surface plasmon resonance sensors, enable the detection of low-abundance biomarkers associated with ovarian tumors [154]. Furthermore, multi-biomarker detection, facilitated by these advanced biosensors, can provide a more comprehensive diagnostic picture, increasing the accuracy of ovarian cancer detection [155]. The integration of microfluidic platforms and artificial intelligence (AI) has further augmented the potential of biosensors in clinical diagnostics. Microfluidic technologies allow for rapid and low-cost analysis, requiring minimal sample volume and providing the capability for point-of-care testing [124]. AI algorithms, when applied to data generated from biosensor devices, can enhance diagnostic accuracy, assist in real-time decision making, and improve prognostic predictions based on complex biomarker patterns [156]. Despite these promising advances, there are still several hurdles in the clinical application of biosensors. Standardization of biosensor technologies, ensuring reproducibility across different platforms, and regulatory approval for clinical use remain key challenges [102]. Additionally, clinical validation of these biosensors is essential to demonstrate their efficacy and reliability in real-world settings, especially when integrated into routine patient care [157]. Future research should focus on refining these technologies, developing robust biomarker panels and ensuring their widespread availability in clinical environments. In conclusion, ovarian tumor-based biosensors present a promising avenue for early detection, monitoring, and treatment of ovarian cancer. With continued advancements in biosensor design, integration with microfluidics, and AI applications, these technologies have the potential to revolutionize ovarian cancer diagnostics, ultimately leading to improved patient outcomes and survival rates.

As discussed above, researchers have made tremendous progress in the field of biosensors for the detection of ovarian cancer biomarkers. Most of the published biosensing methods for OC detection in biological samples do not require expensive equipment and have been demonstrated as proofs of concept to show their practical potential. Despite great advances, most sensing technologies suffer from several drawbacks that make it difficult to translate laboratory research into commercially viable products. For example, the normal range of ovarian cancer markers in body fluids is at the nanoscale level, and their application in routine clinical analysis is highly dependent on their detection with sufficient sensitivity and specificity. Therefore, development of commercial-grade biosensors for the detection of ovarian cancer biomarkers remains a challenging problem. Moreover, the reported biosensing strategies are mainly designed and developed based on pure samples of analytes, free of interfering biomolecules that may significantly affect the results, thus enabling low detection limits and wide concentration ranges of target analytes to be achieved. However, real clinical sample analysis using these biosensors faces many challenges, including cross-reactivity, sample collection, transportation, storage, and application modes, to obtain the same results. Therefore, intensive research is needed to develop commercial-grade miniaturized biosensing technologies for sensitive and selective detection of ovarian cancer biomarkers in a cost-effective manner in routine clinical analysis.

## 9. Conclusions and Future Perspective

The fundamental reason for the early detection of ovarian cancer from its asymptomatic early stages is to control disease progression and improve the prevention through personalized interventions. Ovarian cancer disproportionately affects millions of women worldwide and causes a significant economic burden. In this review, we provided an overview of biomarkers and application of various biosensing modalities for the early detection of ovarian carcinomas. As discussed above, different research groups applied different techniques for the design and development of sensor platforms or their functionalization or surface modifications using nanomaterials. It is well documented these biosensors can efficiently detect ovarian cancer biomarkers, including MUC16 mucin (CA-125), Human Epididymis Protein 4 (HE4), Human Prostasin (PSN), Mesothelin, Osteopontin (OPN), Kallikreins, Heat Shock Proteins (HSPs), MicroRNAs (miRNAs), and Exosomes, in biological fluids in a concentration-dependent manner. These findings offer promising alternatives to conventional methods for early detection and diagnosis of ovarian cancer. Notably, conventional methods require expensive and sophisticated equipment and skilled technical staff to maintain and operate the experiments in order to obtain test results. Thus, biosensor technology is the hope for the future for rapid and early detection of important biomarkers to reduce ovarian cancer lethality, morbidity, and mortality. More importantly, the development of a low-cost, potentially point-of-care-sensing platform would greatly assist patients in self-monitoring by allowing them to determine their own disease progression. Such a strategy would reduce economic losses in both developed and underdeveloped countries and would be more convenient for patients who cannot afford expensive routine medical tests or who do not have proper access to healthcare facilities.

## Figures and Tables

**Table 1:** Recent development in the biosensing methodologies for the sensitive detection of ovarian cancer biomarkers.

Biomarker	Materials Used	Method of Detection	Linear Range	LOD	Reference
CA-125	Antibody-functionalized nanosized GO-	Fluorescence	0.1–10 U/mL	0.01 U/mL	[65]
CA-125	Nanogold thin film doped into a sol−gel matrix	Fluorescence quenching	2.0−127.0 U/mL	1.45 U/mL	[66]
CA-125	Ni-phthalocyanine complex doped in polystyrene matrix	Fluorescence quenching	0.001–127 U/mL	0.0001	[69]
CA-125	Ni and Cd nanoclusters	Fluorescence	0.0005–40 U/mL	50 µU/mL	[70]
CA-125	Carbon Quantum dots entrapped in polymethyl methacrylate (PMMA) matrix	FRET	0.01–129 U/mL	0.66 U/mL	[72]
CA-125	Red-emitting DNA-AgNCs with CA-125 aptamer (rDNA2-AgNCs-apta2)-Target induced AuNPs aggregation and fluorescence recovery from surface plasmon-enhanced energy transfer (SPEET)	Fluorescence	0.01 and 2.0 U/mL	0.015 U/mL	[83]
CA-125	Gold–silver alloy film-based SPR (AuAg-SPR) sensor	SPR	0.1–10 U/mL	0.1 U/mL	[84]
CA-125	Nanogold-functionalized copper–cobalt oxide nanosheets (CuCo-ONSs@AuNPs) as nanocomposites	Electrochemical	1 × 10^−7^ U/mL to 1 × 10^−3^ U/mL	3.9 × 10^−8^ U/mL	[100]
CA-125	Screen-printed carbon electrodes modified with polytoluidine blue (PTB)/AuNps	Electrochemical	5–100 pg/mL	1.20 pg/mL	[101]
CA-125	Hierarchical microporous carbon material fabricated from waste coffee grounds (WCG) modified screen-printed electrode decorated with AuNps	Electrochemical	0.5–50.0 U/mL	0.4 U/mL	[103]
CA-125	Graphitic carbon nitrides/molybdenum disulfide/magnetic nanoparticles (g-C_3_N_4_/MoS_2_/Fe_3_O_4_) immobilized glassy carbon electrodes (GCEs)	Electrochemical	2–10 U/mL	0.215 U/mL	[104]
CA-125	Eu metal–organic framework-loaded isoluminol-Au nanoparticles (Eu MOF@Isolu-Au NPs)/carboxyl-functionalized CdS quantum dots and N-doped porous carbon-anchored Cu single-atom catalyst	Electrochemiluminescence (ECL)	0.005–500 ng/mL	0.37 pg/mL	[106]
CA-125	Carbon ink/carbon dot/zine oxide (C-ink/CD/ZnO)/silver@polypyrrole (Ag@PPy)	Electrochemical	1 ag/mL–100 ng/mL	0.1 fg/mL	[107]
					
CEA	Deal Aptamer labeled with metalloporphyrinic iron-based metal–organic framework, hemin@MIL-88B (Fe)—Apt1 and luminol-Ap2- Fe_3_O_4_@SiO_2_ adsorbed on magnetic carbon nanotubes (MCNTs)	Chemiluminescence	0.01–100 ng/mL	0.0015 ng/mL	[80]
CEA	Green-emitting DNA-AgNCs with CEA aptamer (gDNA1-AgNCsapta1)-Target induced AuNPs aggregation and fluorescence recovery from surface plasmon-enhanced energy transfer (SPEET)	Fluorescence	0.01–0.9 ng/mL	7.5 pg/mL	[83]
CEA	Antibody-functionalized nanosized GO-	Fluorescence	10–100 pg/mL	~1 pg/mL	[65]
platelet-derived growth factor (PDGF)	Aptamers conjugated AuNPs	Salt-induced AuNps aggregation colorimetry	0.01–10 μg/mL	0.01 μg/mL	[82]
					
lysophosphatidic acid (LPA)	Solid supported actin–gelsolin/dye complex	Action-dye displacement by LPA	0–50 µM	5 µM	[68]
lysophosphatidic acid (LPA)	Gelsolin–actin affinity-based system	Electrochemical	0.01–10 µM	0.7 µM	[95]
					
HE4	Antibody-functionalized nanosized GO-	Fluorescence	10–100 pg/mL	~1 pg/mL	[65]
HE4	Antibody-conjugated gold chip coated with cysteamine	Surface plasmon resonance imaging (SPRi)	2–120 pM	2 pM	[86]
HE4	NiFe_2_O_4_ nanotubes (NTs)/Au nanoparticles (ed-Au NPs)/Thiolated captured DNA/Anti HER4 linked complementary DNA	Electrochemiluminescence (ECL)	10^−6^ ng/mL–10 ng/mL	3 × 10^−7^ ng/mL	[91]
HE4	Eu metal–organic framework-loaded isoluminol-Au nanoparticles (Eu MOF@Isolu-Au NPs)/carboxyl-functionalized CdS quantum dots and N-doped porous carbon-anchored Cu single-atom catalyst	Electrochemiluminescence (ECL)	0.005–500 ng/mL	1.58 pg/mL	[106]
APF	Antibody-functionalized nanosized GO-	Fluorescence	10–100 pg/mL	~1 pg/mL	[65]
CYPA	SiO_2_-encapsulated Au star@AgAu yolk shell nanostructure (YSNS)	Surface-enhanced Raman scattering	10^−7^ μg/mL–10^−2^ μg/mL	7.76 × 10^−10^ μg/mL	[90]
MicroRNA (miRNA-21, miRNA-141, and miRNA-200a)	Silicon-on-insulator structures (SOI-NWs)	Electrochemical	1.1 × 10^−17^ M–1.1 × 10^−14^ M	1.1 × 10^−16^ M	[92]
Exosomes	Entropy-driven strand displacement reaction (EDR) process and DNAzymes-induced cleavages	Electrochemical	-	30 particles/μL	[98]
Exosomes	Metal–organic frameworks assembled “double hook”-type aptamer	Electrochemical	31 to 3.1 × 10^6^ particles/μL	12 particles/μL	[94]
mesothelin	Self-assembled monolayer cysteamine chip	Quartz Crystal Microbalance (QCM)	100 pg/mL–50 ng/mL		[118]
